# Solitary Fibrous Tumor of the Lumbar Spine: The Great Mimicker—Report of the Fifth Case

**DOI:** 10.1155/2014/852830

**Published:** 2014-07-09

**Authors:** Amer Sebaaly, Lara Raffoul, Ronald Moussa

**Affiliations:** ^1^Department of Orthopedic Surgery, Hotel Dieu de France University Hospital, Alfred Naccache Street, Achrafieh, P.O. Box 166830, Beirut, Lebanon; ^2^Department of Neurosurgery, Hotel Dieu de France University Hospital, Alfred Naccache Street, Achrafieh, P.O. Box 166830, Beirut, Lebanon

## Abstract

Solitary fibrous tumor (SFT) is a rare neoplasm occurring in the central nervous system. It rarely occurs in the spine. This paper reports the fifth case of SFT in a 34-year-old female and focusses on differential diagnosis and importance of surgical treatment.

## 1. Introduction

Solitary fibrous tumor (SFT) is a rare neoplasm that was initially described in the visceral pleura and subsequently reported in extrapleural sites including the pericardium, peritoneum, mediastinum, lung, upper respiratory tract, thyroid, liver, testicle, nasal cavity, parotid, orbit, and meninges [1–3]. The latter location remains particularly rare with few cases of meningeal SFT (mSFT) reported in the literature [4] and fewer cases of the lumbar spine are even reported. We herein report the fifth case of solitary fibrous of the lumbar spine.

## 2. Case Report

A 32-year-old female, with no significant medical history, has had paresthesia of both lower limbs for one year. This paresthesia was associated with nocturnal lumbar pain decreasing with nonsteroidal anti-inflammatory drugs (suggesting osteoid osteoma). Three months ago, she noted an aggravation of her low back pain which became resistant to all types of medication. She also noted a limitation of her walking distance without any motor symptom or neurologic claudication.

On physical examination, she presented a normal motor exam of both upper and lower limbs. She only had diminution of proprioception in distal right lower limb (no definite dermatome). She had normal osteotendinous reflexes, normal anal sensation, and bilateral absence of Babinski sign.

**Figure 1:**
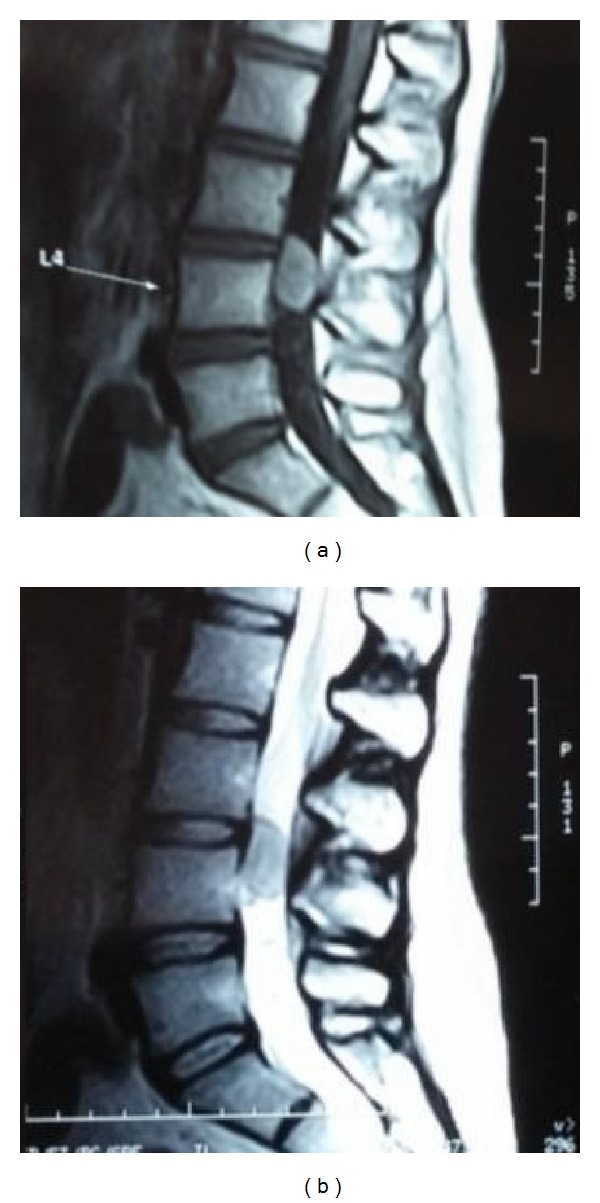
MRI of the lumbar spine showing a hyperintense (T1 weighted images (a)) and hypointense (T2 weighted images (b)) intracanalar tumor on the posterior aspect of the L3-L4 disc.

X-ray did not show any abnormality. An MRI showed a hyperintense lesion of the lumbar spine on T1 weighted images on the posterior aspect of the L3-L4 disc. The lesion is hypointense on T2 weighted images. It showed contrast uptake with gadolinium injection (Figure 1). The differential diagnosis was a lumbar meningioma, schwannoma, spondymoma of the filum, or the less frequent solitary fibrous tumor.

She was operated on with resection of hard, highly vascular tumor in a piecemeal fashion. The tumor had a dural attachment to the anterior part of the dural sac. Her postoperative period was noticeable for disappearance of her symptoms and total recovery. Her follow-up at 16 months is unremarkable.

## 3. Literature Review

The literature review yielded only 4 well-documented studies describing mSFT of the lumbar spine. All characteristics as well as our case are described in Table 1.

## 4. Discussion

SFT was first described in the meninges as a lesion distinct from fibrous meningioma in 1996 [1, 5]. Since then, few cases of mSFT have been reported in both cranial and spinal compartments [4]. Spinal mSFT can arise in the entire spine and accounts for 25% of all mSFT [1] but rarely occurs in the lumbar area (<12%) [9].

Lumbar mSFT presents with the signs of local compression of the conus terminalis, cauda equina, or the exiting nerve roots. Lumbar pain is a frequent sign [1, 6, 9]. On MRI spinal SFTs appear isointense to the spinal cord on T1-weighted images and show variable low signal intensity on T2-weighted images. This latter feature is due to the abundant collagen stroma within the tumor. The contrast enhancement is homogeneous or slightly inhomogeneous [9]. This contract uptake is due to the good blood supply with a prominent mixture of hemangiopericytic blood vessels [6]. MR studies do not differentiate SFTs from other more frequent intraspinal tumors. However, an SFT must be suspected in the presence of an intraspinal lesion with marked hypointensity on T2-weighted sequences [9].

This tumor may mimic other dural-based tumors such as hemangiopericytoma, fibrosarcoma, schwannoma, and neurofibroma, all of which may share pathological similarities with SFT [1] (Table 2). Although not always an easy task, it is extremely important to distinguish mSFT from other meningeal tumors since treatment and prognosis may differ substantially. While postoperative radiation therapy may be indicated for some of the more aggressive tumors such as hemangiopericytoma, mSFT is typically successfully managed by surgical excision alone [1]. While prognosis of mSFT remains unclear given the rarity of this lesion, recurrences and even metastases may rarely occur [10, 11]. Nonetheless, when this tumor shows aggressive behavior, it can be lethal because of its chemoresistance and ability to rapidly metastasize [8]. Of note, 5.8% of central nervous system SFT are malignant and 8% of the reported patients demonstrated ‘‘atypical features” [7]. Thus, malignant features include high cellularity, a high number of mitotic figures, necrosis, and nuclear pleomorphism [10]. Adjuvant radiotherapy is unnecessary [9]. However, most tumors seem to follow a nonaggressive course. It is reasonable to assume that total resection would be the most relevant factor when it comes to minimizing the risk of recurrence [11]. Prognosis of meningeal SFT remains nonetheless unclear and long-term follow-up remains essential for all meningeal SFTs [1].

Histologically, SFT consists of monomorphic spindle cells both organized into straight, curving, or undulating fascicles and arranged in an unstructured fashion. Focally prominent bands of hyalinized collagen are characteristic and spindle cells are typically embedded in a conspicuous fibrous matrix [1]. It is now known that SFT has a mesenchymal origin, on the basis of immunohistochemical studies showing strong and diffuse reactivity for CD34. This is a distinctive feature of SFT, since the majority of meningiomas either do not stain or exhibit only weak focal immunoreactivity for CD34 [6, 11]. The latter is a hialynated transmembrane glycoprotein of unknown function that is found in hematopoietic progenitor cells within the bone marrow, endothelial cells, and some fibroblast-related mesenchymal cells [11]. SFT may actually originate from this latter type of cells. Another pathological criterion for SFT is negative immunoreactivity to epithelial markers, while most fibrous meningiomas are positive for EMA and some of them for S-100 [11].

## Figures and Tables

**Table 1:** 

Author	Age	Localisation	Symptoms	Treatment	Follow-up
Carneiro et al. [5]	F/54	L1-L3	Bilateral leg pain, paresthesia, and weakness	Total removal from dural origin	Died within 7 years
Donnellan et al. [6]	39/M	Not specified	Myeloradiculopathy	Total removal with no dural origin	No recurrence at long-term follow- up
Montano et al. [7]	56/F	L1-L2	Lumbar pain, paresthesia, and sensory loss in her lower limbs	Total lesion removal	No recurrence at 1 year
Nagano et al. [8]	57/F	L4-L5	Radiculopathy	Partial removal with no dural origin	Died within 6 months of presentation
Our case	F/32	L3-L4	Paresthesia Lumbar pain	Total removal from dural origin	No recurrence on latest follow-up

**Table 2:** MSFT and other dural-based tumors: common and distinctive features.

	Common features	Different features
Fibrous meningioma	Imaging Gross appearance Spindle-shaped cells with abundant intercellular collagen	EMA+ and S-100+ CD34− or weak Well-formed desmosomes Genetic features: loss of DNA on chromosome 22, increase in neurofibromatosis type 2
Hemangiopericytoma	Vimentin+ Leu-7+	Aggressive neoplasm with tendency for recurrences and metastases More densely cellular tumor Polygonal cells with oval nuclei and scant cytoplasm
Myofibroblastoma	Benign Derives from meningeal myofibroblasts	Vimentin+++ Smooth-muscle actin+
Fibrosarcoma	Arises in the meninges and sometimes in the intracerebral perivascular tissue	Malignant and aggressive, responds poorly to treatment Anaplastic, mitotically active cells
Schwannoma	Spindle-shaped cells CD34+ in 89% of cases	Antoni A and B histological patterns
